# Porous material engineering through synthesis for smart sensor systems

**DOI:** 10.1038/s41378-025-01156-2

**Published:** 2026-04-08

**Authors:** Se Jin Choi, Sang Yoon Park, Kang Hyeon Kim, Haneol Lee

**Affiliations:** 1 Division of Advanced Materials Engineering, Jeonbuk National University, Jeonju-si, Jeollabuk-do Republic of Korea; 2 Department of JBNU-KIST Industry-Academia Convergence Research, Jeonbuk National University, Jeonju-si, Jeollabuk-do Republic of Korea; 3 Department of Materials Science and Engineering, Gwangju Institute of Science and Technology (GIST), Gwangju, Republic of Korea

**Keywords:** Structural properties, Electronic properties and materials

## Abstract

Porous materials have emerged as a prominent class of functional materials for next-generation sensor platforms due to their exceptionally high specific surface areas, tunable pore architectures, and versatile chemical functionalization capabilities. These characteristics promote enhanced interactions with target analytes through increased adsorption capacity and accelerated diffusion kinetics^1–3^, providing significant advantages for developing sensors with enhanced sensitivity, selectivity, and reduced detection limits^4,5^. This review systematically examines representative categories of porous materials, including metal oxides, polymers, and carbon-based systems, analyzing their synthesis strategies encompassing sol-gel processes, template-assisted methods, three-dimensional printing, and light-material interactions. Fundamental sensing mechanisms enabled by porous architectures are analyzed, including electrical, electrochemical, and optical transduction pathways. The review explores diverse applications in environmental monitoring, biomedical diagnostics, and smart packaging systems, wherein porous material-based sensors demonstrate substantial improvements characterized by accelerated response times, enhanced analyte discrimination, and extended operational stability. This review provides critical insights into design principles and fabrication methodologies that will inform future research and facilitate practical implementation in advanced sensing technologies.

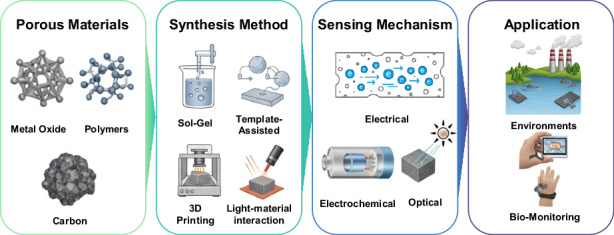

The unprecedented expansion of smart devices and Internet of Things (IoT) technologies has driven an escalating demand for high-performance sensors across diverse application domains, ranging from environmental monitoring and wearable electronics to biosignal detection systems. Modern sensor technologies must transcend conventional performance metrics of sensitivity and selectivity to accommodate the stringent requirements of contemporary applications, including enhanced mechanical flexibility, extended operational longevity, multifunctional capabilities, and rapid dynamic response characteristics. Consequently, the development of advanced functional materials that exhibit exceptional responsiveness to external stimuli while maintaining structural and operational integrity under varying environmental conditions has emerged as a critical research imperative.

Porous materials have attracted considerable scientific interest as promising platforms for next-generation sensor technologies due to their distinctive structural architectures and physicochemical properties. These materials are characterized by hierarchical pore networks encompassing micropores ( <2 nm), mesopores (2-50 nm), and macropores ( >50 nm), which collectively contribute to exceptionally high specific surface areas, efficient mass transport kinetics, and extensive opportunities for targeted chemical functionalization^6^. Such structural features facilitate enhanced analyte-material interactions, resulting in superior sensing performance characterized by improved selectivity, reduced response times, and amplified signal transduction^7–9^.

Despite these inherent advantages, the practical implementation of porous materials in commercial sensor applications faces several significant technical challenges. The fabrication of uniform and reproducible porous architectures remains highly susceptible to process variables, including thermal treatment conditions and solvent evaporation kinetics, compromising manufacturing scalability and product consistency^10–12^. Furthermore, established synthesis methodologies such as template-directed approaches and self-assembly techniques, while offering precise structural control, are generally incompatible with cost-effective large-scale industrial production requirements^13,14^. Additionally, highly porous structures often exhibit inadequate mechanical stability and structural durability, potentially leading to performance degradation during prolonged operational periods^15,16^.

To address these limitations, numerous innovative fabrication and design strategies have been investigated. Three-dimensional (3D) printing technologies provide enhanced architectural control and manufacturing reproducibility, while flash-lamp-assisted synthesis techniques enable rapid, large-area production of porous structures^17,18^. Moreover, hybrid sensor configurations integrating carbon-based nanomaterials with flexible polymeric matrices have demonstrated the ability to maintain structural integrity without compromising sensing responsiveness or sensitivity^19–22^. The successful integration of porous materials into various sensor platforms has facilitated their deployment in real-world applications, including environmental monitoring systems, wearable sensing devices, biomedical diagnostic instruments, and industrial safety monitoring solutions^23–27^. Furthermore, when structural design is realized in a modular way, it allows for facile customization and integration of multiple functional units, providing enhanced versatility and expandability for diverse sensor applications^28,29^.

This review provides a comprehensive and critical assessment of the current state-of-the-art in porous material-enabled sensor technologies (Fig. 1). Through systematic analysis of key design parameters governing sensing performance, we aim to advance fundamental understanding of how porous materials can be strategically engineered and optimized for specific sensing applications. Particularly, this review emphasizes the synthesis of porous materials, purposefully linking them to sensor applications. This approach enables an analysis of research trends in sensor technologies according to various processing methods. Furthermore, we identify remaining technological challenges and discuss future research opportunities to guide the development of robust, high-performance porous material-based sensor platforms suitable for widespread deployment across environmental, biomedical, industrial, and consumer electronics sectors.

**Fig. 1:**
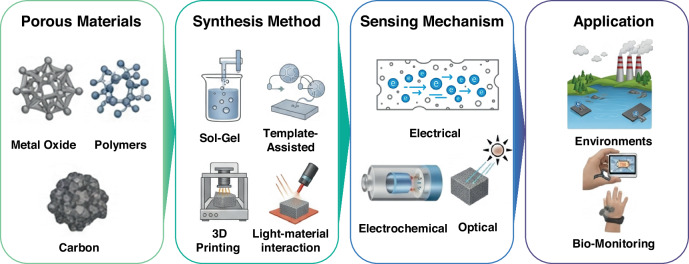
Schematic overview of porous material-based sensing platforms, illustrating representative material classes (metal oxides, polymers, carbon), common fabrication routes (sol–gel, template-assisted assembly, 3D printing, light–material interactions), principal transduction mechanisms (electrical, electrochemical, optical), and key application areas (environmental monitoring, biomedical sensing)

## Porous materials

Porous materials have gained widespread recognition as indispensable functional components in diverse advanced applications, encompassing sensors, energy storage systems, catalysis, and filtration technologies^30–33^. These materials exhibit exceptional specific surface areas and superior mass transport characteristics attributable to their intricate pore architectures, maximizing both physical and chemical interactions with target species. In high-performance sensor applications, porous materials provide distinctive advantages through enhanced analyte-material interactions and precise control over selectivity and sensitivity via strategic surface functionalization^34–36^. The performance characteristics of porous materials are critically dependent upon their compositional properties, which are governed by multiple structural parameters including pore size distribution (micropores, mesopores, and macropores), overall porosity, structural organization, and intrinsic electrical and mechanical properties^37,38^. Among the various material categories, metal oxides, polymers, and carbon-based materials have demonstrated exceptional versatility in sensor platforms due to their structural diversity and functional tunability^39–41^. This section provides a comprehensive examination of these material systems, analyzing the characteristic features of porous architectures formed by each material category and their potential for advanced sensor applications.

### Types of porous materials

#### Metal oxides

Metal oxides represent a class of highly valued inorganic materials for advanced sensor applications, distinguished by their unique electronic structures, exceptional chemical stability, and tunable surface reactivity. Titanium dioxide (TiO_2_) and zinc oxide (ZnO) have emerged as particularly promising candidates for porous material development, attributed to their excellent optical properties derived from wide bandgap characteristics, outstanding chemical durability, and relatively facile structural controllability.

**Fig. 2:**
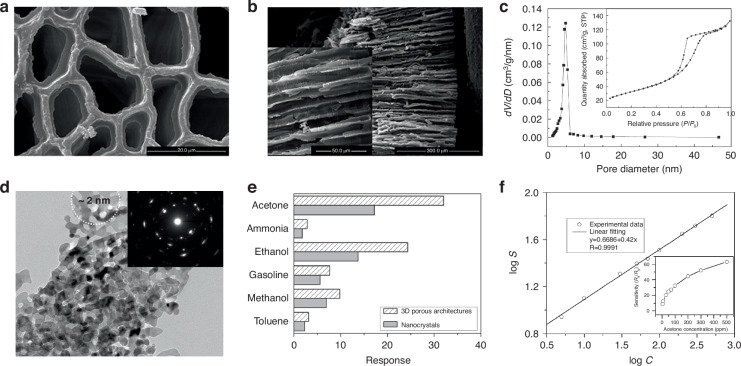
Porous properties in metal oxides. **a** SEM images of the top surface structures of the pearl oyster shell bio-template coated with TiO_2_ precursor gel after thermal treatment at 90 °C. **b** The TiO_2_ sample calcined at 450 °C. **c** N_2_ adsorption–desorption isotherm (inset) and the corresponding pore-size distribution curve. Reproduced with permission from ref. ^41^. Copyright 2018, The Royal Society of Chemical. **d** Magnified TEM image obtained from the dash-marked fringe of the ZnO nanosheet. Inset: corresponding SAED pattern. **e** Responses of the 3D porous ZnO architectures and the ZnO nanoparticles to various gases (the concentration of all gases was 100 ppm). **f** Dilogarithm fit curve of the sensitivity of the sensors to the concentration of acetone. Inset: sensitivities of the gas sensor versus various acetone concentrations. Reproduced with permission from ref. ^42^. Copyright 2010, American Chemical Society

TiO_2_ functions as a prototypical n-type semiconductor oxide that exhibits distinct photophysical properties contingent upon its crystalline phase. Zhao et al. successfully synthesized hierarchically ordered macro-mesoporous TiO_2_ structures by employing nacreous oyster shells and P123 block copolymer as dual templating agents^41^. The resulting anatase-phase TiO_2_ exhibited uniform mesopores of ~4.8 nm diameter and macropores spanning several tens of micrometers (Fig. 2a–c), establishing the structural foundation for enhanced photoreactivity. The anatase crystalline phase demonstrates exceptional surface reactivity and efficient electron-hole separation characteristics, significantly improving sensor response kinetics and sensitivity. Additionally, its thermal stability extending to 450 °C renders it particularly suitable for environmental sensor applications requiring robust thermal durability.

ZnO constitutes another metal oxide system characterized by high ionicity, facile crystallization, and excellent reactivity based on surface defect sites, with widespread application in gas sensing technologies. Fan et al. fabricated multilayered three-dimensional porous ZnO nanosheets through pyrolysis of layered zinc carbonate precursors^42^, yielding porous nanoarchitectures composed of overlapping ultrathin sheets (Fig. 2d–f). The ZnO structures comprise crystal particles with average dimensions of 20 nm and mesopores ranging from 20 to 80 nm, providing substantial surface areas and efficient gas diffusion pathways that contribute to exceptional sensing performance. ZnO demonstrates sensitive responses to various reducing gases through conductivity modulation mechanisms involving reactions with surface oxygen ions (O^−^, O_2_^−^), while the porous architecture maximizes the available active surface area for these interactions.

Metal oxide-based porous materials possess significant structural and functional tunability potential, enabling high-performance characteristics through relatively straightforward processing methodologies. Consequently, these materials are anticipated to maintain their position as central research and application targets in the development of advanced high-performance sensor platforms.

#### Polymers

Polymer materials have found extensive utilization in sensor applications due to their inherent mechanical flexibility, chemical stability, and excellent processability characteristics. Unlike inorganic materials, polymers provide intrinsic softness and elasticity, rendering them particularly suitable for integration into wearable, stretchable, and skin-conformal sensing platforms. The incorporation of porous structures within polymer matrices enhances deformability and compressibility properties, enabling substantial improvements in sensor sensitivity, dynamic range, and recovery behavior.

Polydimethylsiloxane (PDMS) represents one of the most widely employed polymeric materials in porous sensor platforms due to its exceptional biocompatibility, tunable mechanical properties, and superior thermal and chemical stability. Two primary fabrication strategies have been developed for constructing porous PDMS structures: gas foaming and phase separation techniques. These methodologies enable the creation of tailored microstructures that influence critical properties including elasticity, dielectric constant, and electron transport behavior.

**Fig. 3:**
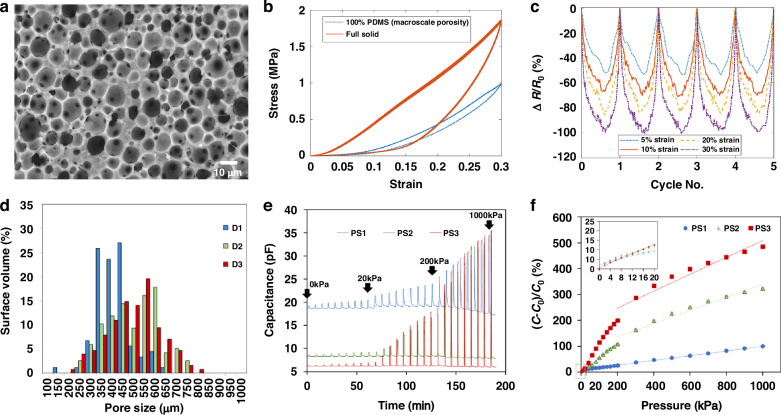
Porous properties in polymers. **a** High-magnification SEM images of the samples with 100% infill and 40% PDMS. **b** Cyclic stress–strain curves for fully solid and 100% PDMS samples. **c** Electrical resistance changes in the five loading–unloading cycles with different maximum strains. Reproduced with permission from ref. ^43^. Copyright 2023, American Chemical Society. **d** Microscopic pore size distribution histograms of the fabricated porous dielectric layers, developed for pressure sensors, by varying the curing temperatures at 110, 140, and 170 °C, Dynamic response (**e**) and corresponding pressure-dependent relative capacitance change (**f**) of porous PDMS-based capacitive pressure sensors. Reproduced with permission from ref. ^44^. Copyright 2021, American Chemical Society

Masihi et al. developed microporous PDMS dielectric materials utilizing an acid-base gas foaming approach with NaHCO_3_ and HNO_3_^43^. The neutralization reaction generates CO_2_ gas, which functions as a template for closed-cell porous network formation within the polymer matrix (Fig. 3a–c). Through systematic control of curing temperature, acid concentration, and PDMS-to-curing agent ratios, the research demonstrated the ability to precisely control pore diameters within the 300–600 μm range and total thicknesses up to approximately 2 mm. The porous structure effectively reduced the dielectric constant while enhancing compressibility, resulting in maximum capacitance changes of approximately 485% under 1 MPa pressure loading. These results demonstrate the effectiveness of chemical foaming methodologies for implementing highly sensitive and compressible polymer dielectrics suitable for capacitive pressure sensing applications.

In an alternative approach, Abshirini et al. utilized evaporation-induced phase separation combined with direct ink writing to fabricate dual-scale porous PDMS/CNT nanocomposites featuring interconnected micro- and macroporous architectures^44^. The microscale pores ( ~ 10-15 μm) originate from phase separation among PDMS, water, and heptane components, while macropores ( >500 μm) are engineered through structural infill patterns implemented during three-dimensional printing processes (Fig. 3d–f). This hierarchical architecture provides mechanical compliance and enhanced piezoresistive response through tunable percolation pathways of conductive CNTs within the polymer matrix. Notably, sensors containing 30% PDMS demonstrated deformation increases up to 900% at 100 kPa and maintained piezoresistive sensitivity over 700 loading cycles, highlighting their durability and long-term reliability for wearable strain sensing applications.

The integration of intrinsic microporosity into polymer frameworks has emerged as a transformative strategy for enhancing electrochemical and functional properties across diverse applications. Porous polymer architectures provide fundamental advantages over their non-porous counterparts, including increased surface area, enhanced mass transport characteristics, and improved structural flexibility that collectively enable superior device performance.

**Fig. 4:**
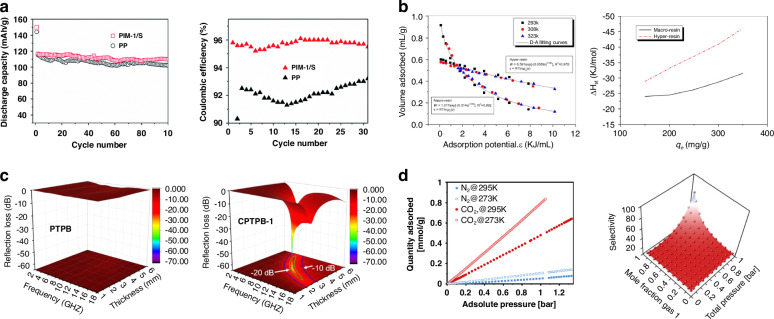
Porous properties in polymers. **a** Discharge capacity and coulombic efficiency comparison of PIM-1/S composite electrode versus conventional PP (polypropylene) separator in Li-ion batteries, showing superior cycling stability and efficiency retention over 100 cycles. Reproduced with permission from ref. ^45^. Copyright 2019, Royal Society of Chemistry. **b** Adsorption isotherms and isosteric enthalpy plots comparing n-hexane vapor adsorption performance between macroporous resin and hypercrosslinked porous resin (Hyper-resin) at different temperatures (293 K, 308 K, 323 K), demonstrating enhanced adsorption capacity and reduced adsorption potential in the hypercrosslinked structure. Reproduced with permission from ref. ^46^. Copyright 2013, Elsevier. **c** Electromagnetic wave absorption performance maps showing reflection loss versus frequency and thickness for non-porous PTPB polymer and porous conjugated microporous polymer CPTPB-1, illustrating significantly improved absorption bandwidth and intensity (−70 dB) in the porous structure at 15 wt% filler loading. Reproduced with permission from ref. ^47^. Copyright 2020, Elsevier. **d** Gas adsorption isotherms comparing N₂ and CO₂ uptake at 273 K and 295 K for functionalized covalent organic frameworks (COFs), along with CO₂/N₂ IAST selectivity map demonstrating enhanced gas separation performance through modulated porous structure design. Reproduced with permission from ref. ^48^. Copyright 2023, Elsevier

Polymers of intrinsic microporosity (PIMs) demonstrate exceptional performance as battery separators and electrode materials, significantly outperforming conventional non-porous polymers (Fig. 4a)^45^. PIM-1/S composite electrodes maintain discharge capacities exceeding 140 mAh/g with coulombic efficiencies above 94% over 100 cycles, representing substantial improvements over traditional polypropylene (PP) separators that exhibit rapid capacity decay and lower efficiency retention. The rigid, contorted molecular structure of PIMs creates permanent free volume elements within the polymer matrix, facilitating efficient lithium-ion transport while simultaneously suppressing polysulfide shuttle effects through enhanced physical barrier properties. This multifunctional capability, combined with superior thermal stability exceeding 300 °C, establishes PIMs as advanced materials for next-generation energy storage systems where both ionic conductivity and mechanical robustness are critical performance parameters.

Hypercrosslinked polymers (HCPs) provide dramatic enhancements in adsorption capacity and kinetics compared to conventional macroporous resins through their permanently microporous network structures (Fig. 4b)^46^. Equilibrium adsorption studies of n-hexane vapor reveal that hypercrosslinked resins achieve 105% higher static adsorption capacity and 43% improved breakthrough performance relative to macroporous analogs at identical operating conditions. Dubinin-Astakhov analysis demonstrates that the hypercrosslinked architecture reduces adsorption potential from ~0.31 kJ/mol to 0.06 kJ/mol, indicating weaker interaction energies that facilitate more rapid adsorption-desorption cycling. Isosteric enthalpy calculations further confirm enhanced thermodynamic favorability, with values decreasing from −45 kJ/mol to −30 kJ/mol as adsorption capacity increases. These performance advantages arise from the combination of ultrahigh specific surface areas (typically 500–2000 m^2^/g) and hierarchical pore structures that provide both rapid diffusion pathways and abundant accessible adsorption sites.

Conjugated microporous polymers (CMPs) exhibit superior electromagnetic wave absorption properties enabled by their π-conjugated frameworks and tunable porosity (Fig. 4c)^47^. Confined polymerization of pyrrole within conjugated polymer networks yields porous materials (CPTPB-1) that achieve reflection losses exceeding −70 dB across broad frequency ranges, compared to maximum values of −50 dB for non-porous analogs (PTPB) at equivalent filler loadings of 15 wt%. The enhanced performance stems from synergistic effects between dielectric and magnetic losses facilitated by the porous architecture, which creates numerous interfaces for electromagnetic wave scattering and absorption. Additionally, the conjugated backbone provides intrinsic conductivity that enables efficient charge transfer and polarization relaxation processes, while the microporous structure contributes impedance matching that maximizes wave penetration into the material. These combined effects enable ultrathin, lightweight electromagnetic shielding materials with absorption bandwidths exceeding 5 GHz.

Covalent organic frameworks (COFs) leverage crystalline porous architectures to achieve exceptional gas separation selectivity through precise control of pore dimensions and surface chemistry (Fig. 4d)^48^. Nitrogen and carbon dioxide adsorption isotherms at 273 K and 295 K demonstrate substantially higher CO_2_ uptake compared to N_2_, with ideal adsorbed solution theory (IAST) calculations predicting CO_2_/N_2_ selectivity exceeding 80 across wide ranges of gas compositions and pressures. The ordered porous structure provides molecular sieving capabilities based on kinetic diameter differences (CO_2_: 3.3 Å, N_2_: 3.64 Å), while functional group modulation of pore walls introduces selective adsorption sites through quadrupole-dipole interactions. This dual-mechanism selectivity enables COFs to outperform traditional porous polymers and zeolites in challenging gas separation applications including post-combustion CO_2_ capture and biogas upgrading. Furthermore, the covalent bonding network ensures exceptional chemical and thermal stability, maintaining performance under industrially relevant operating conditions that would degrade alternative materials.

Porous polymer materials exhibit superior processability, shape adaptability, and mechanical tunability compared to their inorganic counterparts^49,50^. As sensor platforms increasingly demand stretchability and versatility in form factor, polymer-based porous materials are expected to assume progressively critical roles in the development of next-generation flexible and wearable sensors.

#### Carbon-based materials

Carbon-based materials have undergone extensive investigation for high-performance sensor applications due to their inherent lightweight characteristics, superior electrical conductivity, chemical inertness, and versatile nanostructuring capabilities. Among these materials, graphene and carbon nanotubes (CNTs) have emerged as the most prominent candidates due to their exceptional specific surface areas, mechanical flexibility, and excellent charge carrier mobility. These materials can be assembled into porous macroscopic structures that retain desirable nanoscale properties while providing enhanced mechanical compliance and mass transport characteristics.

**Fig. 5:**
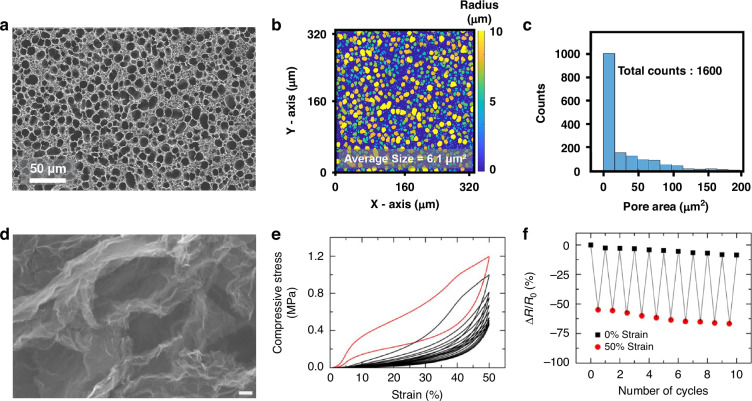
Porous properties in carbon. **a** SEM images of FPG generated under second (at 22 J cm^−2^) flashlight exposures. **b** Pore size distribution of the optimized FPG obtained by consecutive two shots of flash fluence at 22 J cm^−2^. **c** Quantitative pore size distribution of the optimized FPG (twice exposures at 22 J cm^−2^). Reproduced with permission from ref. ^45^. Copyright 2025, John Wiley & Sons. **d** Graphene aerogel with 4 wt% resorcinol–formaldehyde after etching. **e** Compressive stress–strain curves of 10 cycles of loading–unloading. **f** Electrical resistance change when repeatedly compressed up to 50% of strain for 10 cycles. Reproduced with permission from ref. ^46^. Copyright 2015, Springer Nature

Choi et al. reported the fabrication of flash-induced three-dimensional porous graphene via photothermal decomposition of organic precursors^51^. Under flashlamp irradiation fluence of 22 J/cm², the process generated densely packed porous structures with average pore diameters of approximately 60 μm (Fig. 5a). The resulting porous networks exhibited narrow pore size distributions (Fig. 5b), and pore number-area relationships confirmed structural uniformity (Fig. 5c). This architecture provided enhanced compressive elasticity and excellent resistance retention under repeated deformation, demonstrating suitability for multifunctional sensors in wearable electronics applications.

Graphene aerogels have attracted significant attention due to their ultralow densities, mechanical resilience, and high electrical conductivity. Zhu et al. demonstrated periodic graphene aerogel microlattices through direct ink writing of highly viscous graphene oxide inks followed by reduction and carbonization processes. Scanning electron microscopy revealed precisely defined hierarchical architectures integrating microscale lattice filaments with nanoscale pores (Fig. 5d)^52^. Mechanical characterization highlighted their superior stiffness properties, with printed aerogels exhibiting Young’s moduli up to an order of magnitude higher than bulk counterparts of similar density, attributed to local filament densification effects (Fig. 5e). Piezoresistive stability was validated through resistance variation monitoring under 50% cyclic strain, demonstrating highly consistent behavior over repeated cycles (Fig. 5f). Beyond mechanical robustness, the aerogels maintained high surface areas ( ~ 1100 m²/g) and conductivities exceeding 200 S/m, both essential characteristics for reliable piezoresistive sensing applications.

These approaches exemplify the versatility of carbon-based porous architectures. Flash-induced graphene provides ultrafast and scalable fabrication suitable for wearable device integration, while direct ink written aerogels offer precise structural control and superior mechanical reinforcement. Their complementary advantages highlight the potential of engineered porous carbon systems for next-generation multifunctional sensors and flexible electronics.

### Synthesis methods

The synthesis of porous materials plays a critical role in determining their structural and functional properties, directly influencing their suitability for high-performance sensor applications^53,54^. Numerous synthetic strategies have been developed to precisely control pore size, morphology, surface chemistry, and mechanical integrity, enabling customization of material performance for specific operational environments^55–58^. Among the most frequently utilized methods are sol-gel processes, template-assisted synthesis, three-dimensional printing, and light-material interactions^59–61^. Sol-gel methodologies enable molecular-level control of network formation, facilitating straightforward incorporation of dopants or functional moieties during synthesis. Template-assisted approaches, employing either soft templates (surfactants, block copolymers) or hard templates (silica spheres, polymer beads), provide highly ordered and uniform pore structures with tunable dimensions across microporous, mesoporous, and macroporous regimes. Three-dimensional printing has emerged as a transformative approach, enabling direct digital design and fabrication of hierarchical porous structures with customized geometries, effectively bridging nanoscale structural control with device-scale integration. Light-material interactions, including CO_2_ laser and flashlamp irradiation, provide ultrafast and energy-efficient processing pathways that produce three-dimensional porous frameworks with improved electrical conductivity and mechanical resilience within milliseconds. These synthetic methodologies offer distinct advantages in structural precision, scalability, and compatibility with various material systems, playing crucial roles in the development of porous materials for practical and multifunctional sensor applications.

#### Sol-gel method

The sol-gel method represents one of the most versatile and widely adopted strategies for porous material synthesis, enabling molecular-level control over network formation and pore architecture^62–64^. Traditional sol-gel processes involve hydrolysis and condensation of metal alkoxides in solution, often assisted by surfactants or block copolymers as structure-directing agents. This approach enables preparation of mesoporous oxides with adjustable pore sizes, high surface areas, and uniform structures. However, conventional wet sol-gel processes typically require substantial quantities of organic solvents, involve slow evaporation-driven assembly, and necessitate time-intensive drying steps, limiting their scalability and environmental sustainability^65^.

**Fig. 6:**
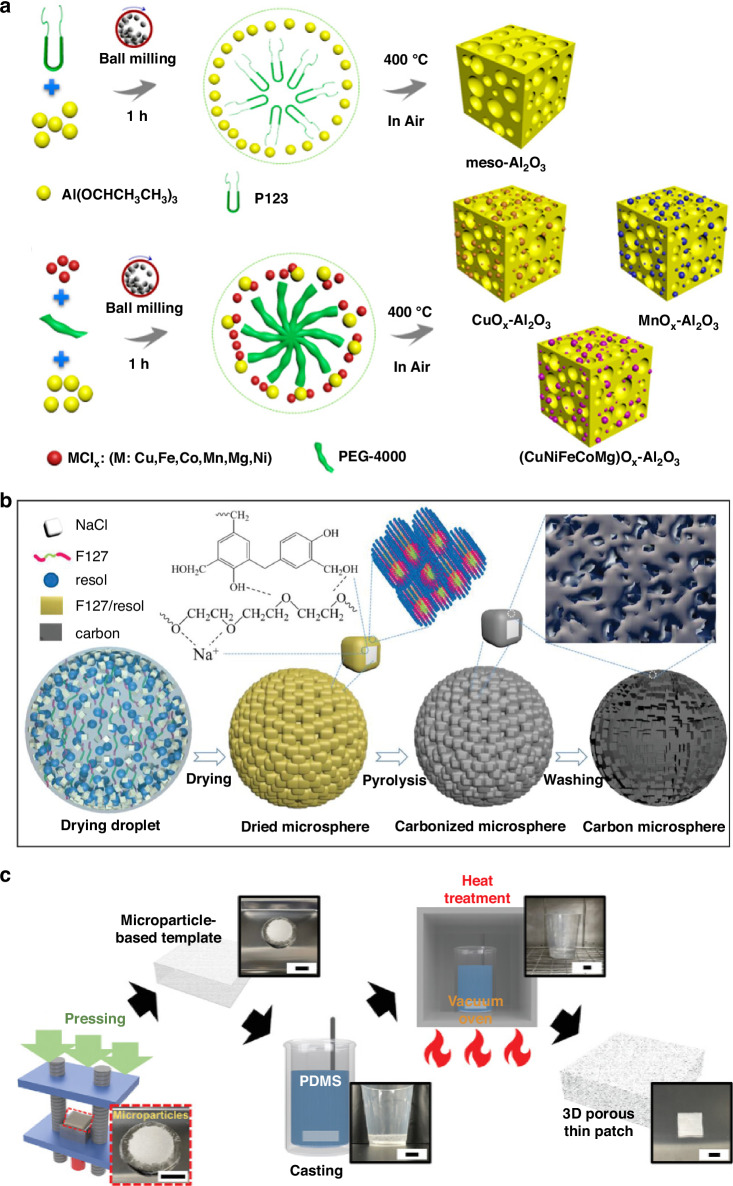
Method for making porous materials (sol-gel, template assisted). **a** Synthesis of meso-Al2O3, Mesoporous Mixed Metal Oxides Using the Mechanochemical NHSG Method. Reproduced with permission from ref. ^66^. Copyright 2019, American Chemical Society. **b** Schematic illustration of the structure assembly mechanism of the HPCMs. Reproduced with permission from ref. ^71^. Copyright 2023, John Wiley & Sons. **c** 3D images and optical images of the porous patch fabrication processes (scale bar = 2 cm). Reproduced with permission from ref. ^72^. Copyright 2023, John Wiley & Sons

Recent advances have introduced nonhydrolytic sol-gel (NHSG) methods and mechanochemical strategies to address these limitations. Zhang et al. developed a mechanochemical NHSG method integrating ball milling with soft-template-assisted assembly to synthesize mesoporous Al_2_O_3_ and multimetallic oxide hybrids in solid-state conditions (Fig. 6a)^66^. In this process, aluminum isopropoxide and polymeric templates (Pluronic P123, PEG) are intimately mixed under mechanical force, enabling rapid polymer-precursor assembly within 60 minutes. Subsequent calcination produces mesoporous Al_2_O_3_ with exceptionally high surface areas (up to ~644 m² g⁻¹) and narrow pore size distributions ( ~ 5 nm). Furthermore, incorporation of anhydrous metal chlorides into the milling process enables synthesis of binary and high-entropy multimetallic oxides ((CuNiFeCoMg)Ox-Al_2_O_3_) while maintaining mesoporosity and structural uniformity. This mechanochemical sol-gel method eliminates solvent usage and significantly reduces synthesis time compared to conventional sol-gel approaches, highlighting its potential for scalable fabrication of porous materials for sensor platforms.

#### Template-assisted synthesis

Template-assisted synthesis provides a straightforward yet highly effective methodology for constructing porous materials with well-organized structures. Unlike purely solution-driven processes such as sol-gel methods, this strategy relies on pre-definition of pore structures using sacrificial templates that function as structural scaffolds during material formation^67,68^. Template-assisted synthesis is generally classified into hard-template and soft-template approaches, depending upon whether rigid colloids (silica particles, salt crystals, metal-organic frameworks) or flexible molecular assemblies (surfactants, block copolymers) are employed. Following template removal, the resulting frameworks exhibit highly uniform pore distributions and interconnected channels, characteristics particularly beneficial for sensor applications where rapid analyte diffusion and maximized interfacial interactions are critical^69,70^.

Li et al. reported the synthesis of three-dimensional carbon microspheres with maze-like mesoporous structures using spray-drying processes combined with NaCl microparticle templating and F127/resol assembly. During drying, the resol-block copolymer composite self-assembled around salt particles, which were subsequently removed through pyrolysis and washing, yielding hierarchical porous carbon spheres with large mesopore tunnels (Fig. 6b)^71^. These structures maximize available surface area and facilitate rapid ion and electron transport, demonstrating significant potential for electrochemical and sensor applications.

Kim et al. developed microparticle-templated three-dimensional porous PDMS patches for long-term wearable UV sensors^72^. Sugar microparticles were initially compacted into laminated molds, infiltrated with PDMS prepolymer, and subsequently cured. Following sugar template dissolution, sponge-like PDMS patches with uniformly interconnected micropores were obtained (Fig. 6c). This porous substrate enabled sweat permeability and metabolic by-product removal from skin surfaces, significantly enhancing stability and comfort of wearable optoelectronic devices.

Template-assisted methods provide powerful approaches for producing porous materials with highly tunable and reproducible structures. Through appropriate selection of template types and removal strategies, researchers can design porous frameworks with optimized physical, chemical, and mechanical properties, expanding their applicability across diverse sensor platforms.

#### 3D-printing

Three-dimensional printing has emerged as a powerful tool for producing porous materials with customized architectures that are challenging to achieve using conventional synthesis methods. Three-dimensional printing enables precise spatial control of pore distribution and structural hierarchy, effectively bridging nanoscale material functionality with macroscale device integration^73,74^. Among various printing techniques, digital light processing (DLP) and direct ink writing (DIW) have been extensively studied for porous material fabrication. While DIW relies on extrusion of particle-based inks, DLP provides significantly higher resolution through localized photopolymerization, enabling rapid fabrication of complex geometries without supporting materials^75^.

**Fig. 7:**
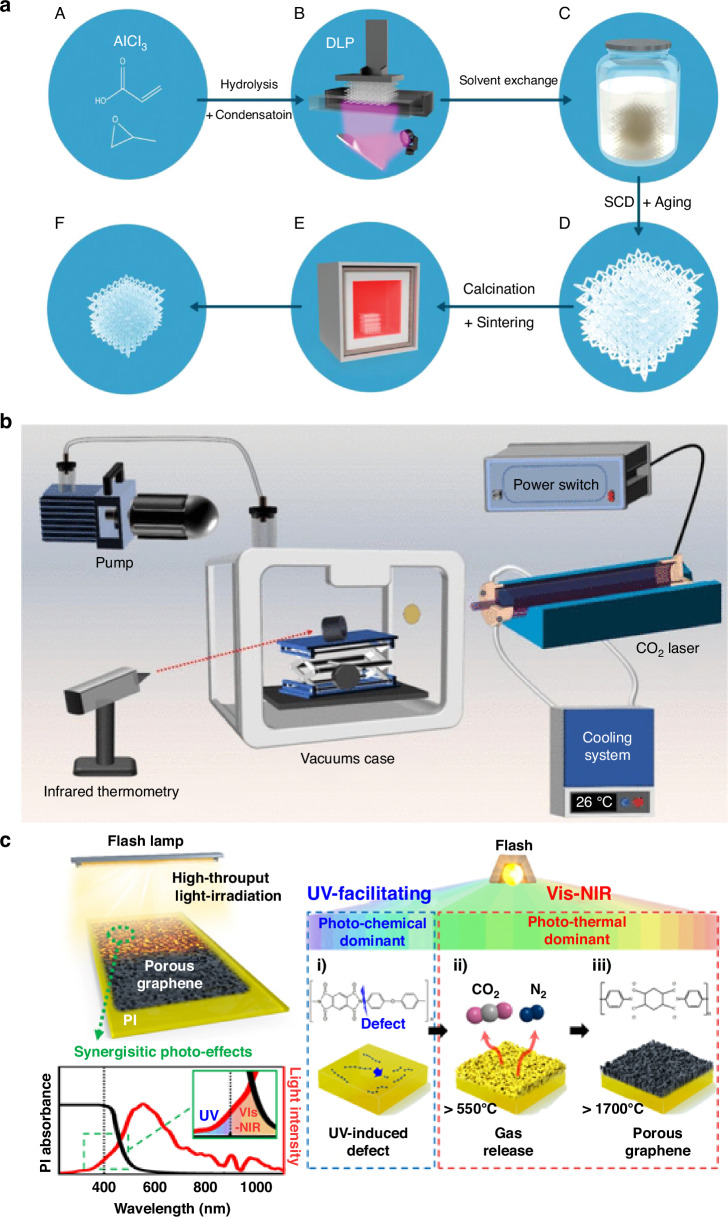
Method for making porous materials (3D printing, Light-material interaction). **a** Scheme of the process for fabricating 3D transparent alumina by DLP printing. Main components of the printing composition; DLP printing; solvent exchange stage; 3D-printed structure after SCD; sintering; final structure. Reproduced with permission from ref. ^76^. Copyright 2023, John Wiley & Sons. **b** Self-assembly laser-induced devices. Reproduced with permission from ref. ^79^. Copyright 2025, American Chemical Society. **c** Schematic of the photo-pyrolysis process, facilitated by synergistic photo-effects and its application. The fabrication process can be broadly divided into three steps: (i) UV-induced photochemical reactions, (ii) Vis–NIR-induced photothermal reactions, and (iii) porous graphene synthesis. Reproduced with permission from ref. ^17^. Copyright 2023, Springer Nature

Moshkovitz et al. demonstrated fabrication of transparent three-dimensional γ-alumina porous structures through integration of sol-gel chemistry with DLP-based three-dimensional printing^76^. In this process, aluminum chloride and propylene oxide facilitated hydrolysis and condensation reactions, resulting in photocurable sol-gel inks (Fig. 7a). The inks were polymerized under patterned UV irradiation to form three-dimensional architectures, followed by solvent exchange and supercritical drying to preserve porous frameworks without cracking. Finally, calcination and sintering at ~850 °C produced crystalline γ-Al_2_O_3_ monoliths with exceptionally high surface areas ( >1800 m² g⁻¹) and optical transparency exceeding 80% at 600 nm. Importantly, shrinkage occurring during drying and sintering improved effective resolution, enabling printed features as small as approximately 6 μm, significantly exceeding the nominal resolution of commercial DLP printers employed.

This strategy emphasizes the unique advantage of three-dimensional printing in porous material synthesis, specifically the ability to integrate designed macroporosity defined by CAD models with intrinsic micro- and mesoporosity resulting from sol-gel chemistry. Consequently, this method enables scalable fabrication of porous ceramics with customized optical, thermal, and mechanical properties. These three-dimensional printed porous frameworks demonstrate significant potential for advanced sensing platforms where precise structural control, high surface accessibility, and integration into complex device geometries are essential.

#### Light–material interaction

Light-material interactions provide unique methodologies for engineering porosity directly within solid frameworks by harnessing photon-material interactions. Rather than relying on bulk thermal treatment or templating strategies, this approach utilizes localized photochemical bond cleavage and photothermal decomposition to generate hierarchical pores within specific regions^77,78^. The outcomes can be precisely controlled through irradiation parameters including wavelength, pulse duration, and fluence, enabling rapid processing under ambient conditions. These characteristics render light-material interactions particularly attractive for producing porous materials that provide both structural complexity and compatibility with flexible substrates and device integration.

Ma et al. reported fabrication of laser-induced porous graphene (LIG) using CO_2_ infrared lasers, which directly convert carbon-rich precursors into conductive porous graphene networks^79^. Intense localized heating during laser irradiation breaks C-O, C-N, and C-H bonds, while simultaneous release of gaseous species generates mesoporous and macroporous channels within graphitic walls (Fig. 7b). The resulting structures exhibit large specific surface areas, efficient ion/electron transport, and high electrical conductivity, properties demonstrated to enhance lithium-ion battery performance and extensible to sensing applications requiring high sensitivity and rapid response.

In addition to laser-based methods, flashlamp irradiation has been employed as an effective fabrication approach for inducing porosity in carbon frameworks. Lee et al. reported a flash-induced high-throughput method wherein broadband xenon flashlamp irradiation simultaneously provides UV and visible-NIR components, generating synergistic photo-effects^17^. UV photons initiate bond scission and defect formation in polyimide substrates, enhancing light absorption, while visible-NIR photons induce rapid photothermal heating, gas evolution, and carbonization (Fig. 7c). This sequence produces hollow-pillared porous graphene with tunable pore sizes (ranging from approximately 1.4 to 19.2 μm as fluence increases from 18 to 24 J cm⁻²), ultralow density ( ~ 0.035 g cm⁻³), and low sheet resistance ( ~ 18 Ω sq⁻¹). Importantly, the method achieves uniform large-area films up to 10 × 10 cm² in single pulses, representing more than 60-fold productivity increases compared to conventional laser-induced methods.

From sensing perspectives, light-material interactions provide unique advantages by directly adjusting porosity and surface chemistry to optimize device performance. The hierarchical pores generated by photochemical and photothermal processes enhance analyte accessibility, accelerate diffusion kinetics, and increase active site density, collectively contributing to enhanced sensitivity and faster response times. Additionally, the capability to pattern porous architectures on flexible substrates under ambient conditions enables seamless integration into wearable and portable devices, where breathability, mechanical compliance, and stability are essential. Specifically, its ability to directly synthesize porous materials on flexible substrates makes it the most suitable process for developing wearable devices. These combined advantages highlight light-induced porous materials as promising platforms for multifunctional sensors demanding both superior performance and adaptability to diverse operating environments.

## Sensing mechanisms of porous materials

The sensing mechanisms of porous materials originate from their distinctive structural and physicochemical properties, which fundamentally govern their interactions with external stimuli, including gas molecules, ions, photons, mechanical stress, and biological species. The exceptionally high specific surface areas provide numerous active sites for adsorption and molecular interactions, while interconnected pore networks facilitate rapid diffusion and mass transport processes^80,81^. These characteristics enhance signal transduction by ensuring that minimal variations in the local environment produce measurable changes in the material’s electrical, electrochemical, or optical properties. Furthermore, the tunable pore size distributions, surface chemistry modifications, and hierarchical architectures enable customization of sensing responses for specific analytes or stimuli. Micropores maximize adsorption capacity for gas detection applications, mesopores promote ionic mobility for electrochemical sensing systems, and macropores enable light scattering or multiple reflections beneficial for optical signal amplification^82,83^. Porous materials function as versatile platforms where structural design is directly integrated with transduction mechanisms, enabling highly sensitive, selective, and multifunctional sensing capabilities across diverse applications.

### Electrical sensing mechanisms

Electrical sensing in porous materials exploits the interactions between external stimuli and charge transport processes throughout the porous framework. Unlike dense planar structures, porous architectures provide enhanced compressibility, substantially larger effective surface areas, and modified interfacial electric fields, which synergistically amplify sensor responses^84–87^. In capacitive-type pressure sensors, the incorporation of porosity into dielectric layers significantly modifies device performance characteristics.

**Fig. 8:**
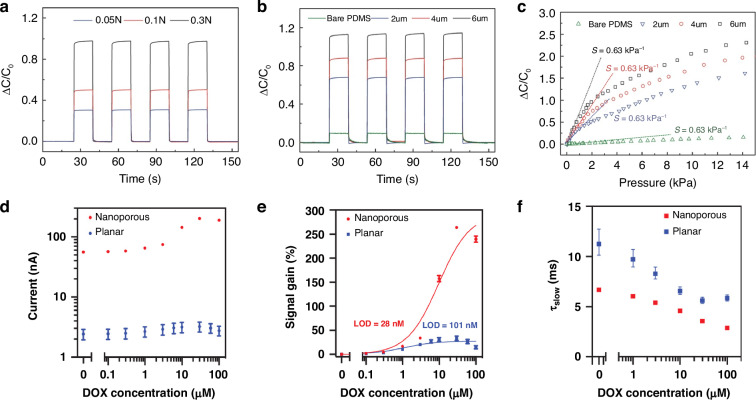
Research on improving the electrical properties of porous materials. **a** Response (capacitance variation) of the porous structured pressure sensor with 4 µm pores for the various applied loads of 0.05, 0.1, and 0.3 N. **b** Response of the porous structured pressure sensors with different pore sizes of 2, 4, and 6 µm to a force of 0.2 N. **c** Relative capacitance curves for the three different porous structured pressure sensors and the unstructured pressure sensor against increasing pressure. The maximum slope of relative capacitance change of the pressure sensor is 0.63 kPa^−1^ (for 6 µm pores). Reproduced with permission from ref. ^88^. Copyright 2016, John Wiley & Sons; **d** Signal level and **e** signal gain of electrochemical aptamer sensors employing nanoporous (red) and planar (blue) electrodes in the presence of 100 × 10^−9^_M_ – 100 × 10^−6^_M_ DOX. Plots are averaged over three replicates. Error bars represent the standard deviation. **f** The decay time (τ_slow_) of both electrodes at various DOX concentrations. Error bars represent the confidence intervals of the curve fitting. Reproduced with permission from ref. ^89^. Copyright 2021, John Wiley & Sons

Kang et al. demonstrated that porous PDMS layers exhibit superior elasticity and adjustable dielectric permittivity compared to bulk PDMS counterparts^88^. Under applied external pressure, two primary mechanisms contribute to capacitance changes: (i) reduction of electrode separation due to pore collapse, and (ii) increase in effective dielectric constant as the air volume fraction decreases and PDMS contribution dominates. The capacitive response of porous dielectric sensors demonstrates several significant advantages over nonporous structures. The porous PDMS layers produce consistent and noise-free capacitance variations under repeated loading cycles, demonstrating reliable electrical transduction (Fig. 8a). Moreover, the response characteristics can be tailored through pore size control, with larger pores producing more pronounced capacitance variations under identical forces due to their higher compressibility (Fig. 8b). Sensitivity analysis reveals that porous sensors achieve maximum sensitivity of 0.63 kPa⁻¹ in low-pressure regimes, substantially exceeding the 0.08 kPa⁻¹ sensitivity of nonporous structures (Fig. 8c).

In biosensing applications, electrical signals originate from electron transfer processes occurring at electrode-electrolyte interfaces. Nanoporous electrodes provide substantial advantages by enlarging electroactive surface areas and modifying local electric double layer configurations. Fu et al. demonstrated that nanoporous gold electrodes accelerate faradaic electron transfer by reducing charge screening within nanoscale pores, effectively increasing the Debye volume^89^. This structural effect positions redox reporters in aptamer-based sensors within stronger electric fields, improving electron transfer efficiency. The result is significant enhancement in signal amplitude and reduced detection limits relative to planar electrodes. For doxorubicin (DOX) detection using nanoporous electrodes, approximately 24-fold increases in signal amplitude and detection limit reductions from approximately 101 nM to 28 nM were achieved. Furthermore, response times (τslow) for faradaic processes were consistently shorter, indicating accelerated charge transfer kinetics within porous architectures. Nanoporous electrodes demonstrate enhanced current responses (Fig. 8d), increased signal gains (Fig. 8e), and accelerated electron transfer dynamics (Fig. 8f), highlighting the critical role of nanoporosity in amplifying electrochemical transduction pathways.

### Electrochemical sensing mechanisms

Electrochemical sensing operates through interfacial charge transfer processes, wherein sensitivity and selectivity are predominantly determined by target analyte accessibility to electrode surfaces and electron transport efficiency. Porous materials provide unique platforms for optimizing these processes by integrating high surface areas, controlled pore hierarchies, and tailored surface chemistry modifications^90–95^.

**Fig. 9:**
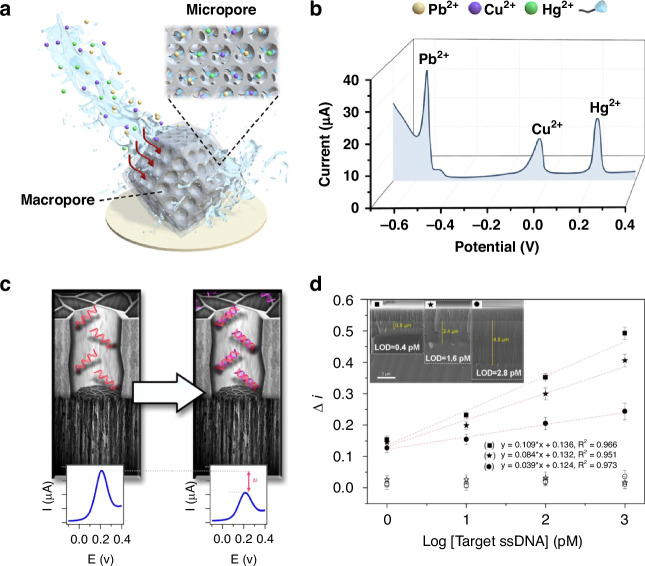
Research on improving the electrochemical properties of porous materials. **a** Schematic diagram of a novel amino-functionalized hierarchical porous carbon prepared by template bonding using ZIF-8 as a precursor and polystyrene spheres as a template. **b** Graph showing excellent electrochemical performance for simultaneous detection of HMI (0.62 nM for Pb^2+^, 1.8 nM for Cu^2+^, and 0.85 nM for Hg^2+^). Reproduced with permission from ref. ^95^. Copyright 2023, American Chemical Society. **c** Schematic of the sensing mechanism of the voltammetric DNA sensor designed using a TCpSi-THCpSi double-layer nanostructure. Plots are averaged over three replicates. Error bars represent the standard deviation. **d** Dose–response curves for the detection of a 28-nucleotide ssDNA in Tris buffer using TCpSi-THCpSi double layer-based biosensors prepared with either specific ssDNA capture probes (solid symbols) or nonspecific ssDNA capture probes as controls (hollow symbols). Reproduced with permission from ref. ^96^. Copyright 2022, American Chemical Society

Hierarchical porous carbons derived from metal-organic frameworks exemplify these advantages. The coexistence of macropores and micropores promotes rapid diffusion of ionic species and provides abundant electroactive sites for redox reactions. The hierarchical structures form interconnected channels that enable efficient mass transport and reduce ion accumulation effects (Fig. 9a). This structural feature produces distinct and well-separated current peaks for Pb²⁺, Cu²⁺, and Hg²⁺ detection (Fig. 9b), demonstrating enhanced selectivity and sensitivity compared to nonporous or purely microporous electrodes^95^.

Porous architectures are instrumental in nucleic acid biosensing applications. Porous silicon electrodes stabilized by layered carbon structures (Fig. 9c) offer expanded effective surface areas for probe immobilization and enhanced conductivity to facilitate electron transfer processes. Upon hybridization with target DNA, conformational and electronic alterations of surface-bound probes result in distinct electrochemical signals, evidenced by voltammetric shifts. Quantitative analysis demonstrates that hierarchical porosity significantly reduces detection limits, enabling sensitivity at femtomolar levels (Fig. 9d)^96^. This performance enhancement is attributed to increased probe loading capacity and improved electric double-layer environments within nanoscale pores, which facilitate acceleration of faradaic reactions.

### Optical sensing mechanisms

Optical sensing employing porous materials capitalizes on their capacity to modulate light-material interactions within both ordered and disordered porous structures. Through integration of structural features including pore size distributions, refractive index modulation, and surface functionalization, porous frameworks can transduce molecular binding events into detectable optical signals, encompassing reflectance shifts, fluorescence intensity changes, or colorimetric responses^97–99^.

**Fig. 10:**
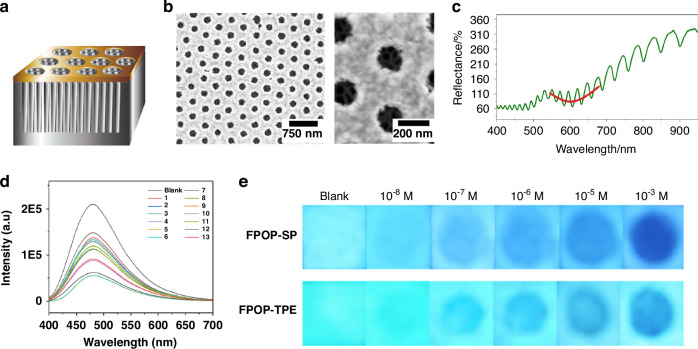
Research on improving the optical properties of porous materials. **a** Schematic of the hybrid sensor. **b** Scanning electron micrographs of the hybrid sensor. **c** Reflectance spectrum of the hybrid sensor recorded in air. Reproduced with permission from ref. ^100^. Copyright 2021, American Chemical Society. **d** Fluorescence spectra [FPOP-SP] = 3 mg/mL (ethanol/water = 90/10) [Nitroaromatic compounds] = 2 × 10^–5^ M. ex = 365 nm, ex/em slits = 5/5 nm. **e** After the test paper containing FPOP-SP and FPOP-TPE compounds was spotted with different concentrations of nitroaromatic compounds 3, pictures were taken under 365 nm UV light. Reproduced with permission from ref. ^103^. Copyright 2022, Springer Nature

Porous silicon decorated with plasmonic nanohole arrays exemplifies this principle. Vertically aligned pore networks (Fig. 10a) provide high refractive index contrast and periodic nanostructuring (Fig. 10b), resulting in distinct photonic resonances observable in reflectance spectra (Fig. 10c)^100^. When analytes permeate the pores, local refractive index changes alter resonance conditions and produce measurable spectral responses. These structures function as highly sensitive transducers capable of detecting subtle variations in molecular adsorption and fluid composition, attributed to pronounced light confinement within nanoscale voids^101,102^.

Fluorescent porous organic polymers (FPOPs) represent another versatile optical sensing platform, combining intrinsic luminescence properties with microporous backbone structures^103^. The extended π-conjugated frameworks of FPOPs enable strong fluorescence emission, which can be quenched upon interaction with electron-deficient analytes such as nitroaromatic compounds. Incremental addition of target molecules results in progressive fluorescence intensity quenching, indicating analyte-polymer interactions at pore surfaces (Fig. 10d). Furthermore, fluorescent test paper development (Fig. 10e) demonstrates practical applicability by enabling visual detection at concentrations as low as 10⁻⁸ M under UV illumination.

Collectively, porous materials enable diverse sensing mechanisms through integration of unique structural features with fundamental transduction principles of electrical, electrochemical, and optical sensing devices.

## Application

The applications discussed in this section were chosen to highlight how innovative porous architectures and material designs are engineered to solve specific challenges rather than solely to catalog the highest performance parameters reported such as multifunctionality, mechanical robustness, and long-term stability.

### Environmental monitoring

Porous materials have emerged as critical components in environmental monitoring applications due to their exceptionally high specific surface areas, hierarchically organized pore networks spanning microporous to macroporous regimes, and versatile surface functionalization capabilities that collectively enable rapid mass transport, selective adsorption, and catalytic degradation of environmental pollutants^62,104–107^. The interconnected porosity facilitates efficient fluid penetration and analyte diffusion processes, while adjustable pore size distributions provide molecular sieving capabilities essential for selective contaminant removal.

**Fig. 11:**
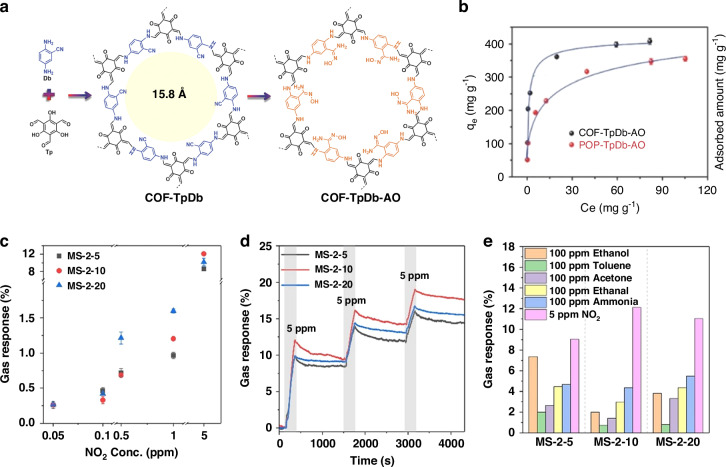
Research on the development of a device for environmental monitoring using porous materials. **a** Synthetic scheme of COF-T_p_D_b_ through the condensation of Tp (black) and Db (blue) and corresponding chemical transformation from the cyano to amidoxime group. **b** Comparison of uranium sorption for COF- and POP-based sorbents. Reproduced with permission. Reproduced with permission from ref. ^108^. Copyright 2022, John Wiley & Sons. **c** Gas response of sensors based on MS-2-5, MS-2-10, and MS-2-20 depending on NO_2_ concentration. **d** Real-time resistance curve of MS-2-5, MS-2-10, and MS-2-20 in response to 5 ppm of NO_2_ for three consecutive times. **e** The maximum resistance change rate of sensors based on MS-2-5, MS-2-10, and MS-2-20 to 100 ppm of ethanol, acetone, ethanol, toluene, NH_3_, and 5 ppm of NO_2_. Reproduced with permission from ref. ^109^. Copyright 2022, Springer Nature

Covalent organic frameworks (COFs) exemplify the potential of porous materials for heavy metal remediation. A COF constructed via Schiff-base condensation of 2,6-diaminopyridine (Db) and 1,3,5-tris(4-formylphenyl)benzene (Tp) yields an ordered hexagonal network with uniform 1.58 nm pores (Fig. 11a). Post-synthetic oxidation converts surface amines into oxime groups (COF-TpDb-AO), dramatically enhancing Pb²⁺ chelation capacity. Adsorption isotherms demonstrate that COF-TpDb-AO achieves lead uptake exceeding 400 mg g⁻¹ at 100 mg L⁻¹ equilibrium concentration, representing 30% higher capacity than unmodified COF, while reaching equilibrium within 30 minutes (Fig. 11b). This performance illustrates the synergistic effects of hierarchical porosity and tailored binding sites for rapid, high-capacity heavy metal removal^108^.

Three-dimensional crumpled Ti_3_C_2_Tₓ MXene spheres produced by aerosol spray drying demonstrate the versatility of porous architectures for gas sensing applications (Fig. 11c–e). The hierarchical macro- to microporous architectures of variants MS-2-5, MS-2-10, and MS-2-20, with progressively higher pore volumes, exhibit NO_2_ sensing responses up to 12% at 5 ppm concentration, with response and recovery times below 20 seconds (Fig. 11d) attributed to accelerated gas diffusion within interconnected porous networks. Selectivity testing confirms greater than 3:1 discrimination of NO_2_ over ethanol, toluene, acetone, and ammonia at 100 ppm (Fig. 11e), while the compressible spherical structures function as piezoresistive pressure sensors capable of resolving light touch and shear forces through distinct resistance changes ( ~ 34 ms response time)^109^.

These applications demonstrate the fundamental advantages of porous materials in environmental monitoring systems. The interconnected pore networks enable rapid mass transport and efficient pollutant capture, while ultrahigh specific surface areas provide abundant active sites for adsorption or catalytic processes. Precise control over pore size distributions and surface chemistry modifications enables tailored selectivity toward specific contaminants, ranging from heavy metals to volatile organic compounds. Moreover, well-designed porous frameworks maintain structural integrity under dynamic environmental conditions and can be regenerated for multiple operational cycles. Scalable and environmentally sustainable fabrication methodologies further support the deployment of porous sorbents and sensors in water purification and air quality monitoring systems.

### Biomedical and health monitoring

Porous material-based wearable sensors have emerged as transformative technologies for real-time health monitoring applications, providing unprecedented capabilities for non-invasive physiological assessment and continuous healthcare surveillance^110–112^. The integration of advanced porous architectures with flexible electronic systems enables the development of robust, durable devices that maintain high performance under mechanical deformation while providing accurate biomarker detection in complex biological environments.

**Fig. 12:**
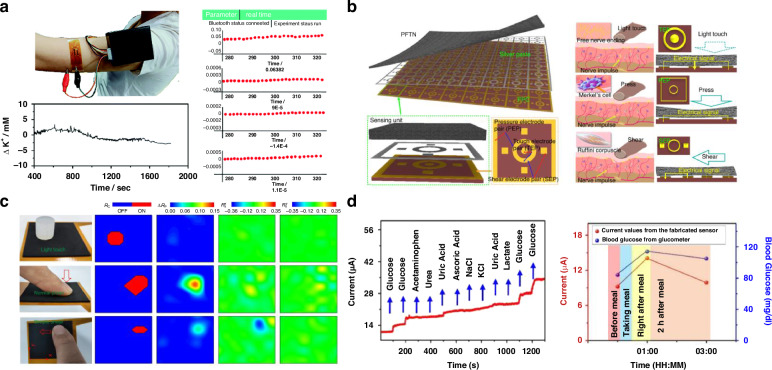
Research on the development of a device for bio-medical and health monitoring using porous materials. **a** A comprehensive figure illustrating the real-time on-body analysis of human perspiration during jogging exercise, including a photograph of an individual wearing a portable electrochemical workstation with a flexible K^+^ sensor and the corresponding real-time curve showing the change in potassium ion concentration in sweat throughout the exercise. Reproduced with permission from ref. ^113^. Copyright 2022, The Royal Society of Chemical. **b** Schematic diagram of the E-skin comprised of an array of sensing units (left bottom) containing PFTN, silver paste, and FPC (right bottom). Biomimetics of the E-skin: TEP simulating free nerve ending (right top), PEP simulating marker cell (right middle), and SEP simulating Ruffini corpuscle (right bottom). **c** Images and contours (Rc, Rp, Rsx, Rsy) of the E-skin under different loading states: light touch of soft silicone (top), normal pressure (middle) and negative x-directional shear (bottom). Reproduced with permission from ref. ^114^. Copyright 2024, Springer Nature. **d** Amperometric response of the flexible porous graphene-based biosensor to the sequential addition of multiple analytes (glucose, acetaminophen, urea, uric acid, ascorbic acid, KCl, NaCl, lactate) and selectivity analysis for glucose in human perspiration. Blood glucose tracking capabilities are further validated by comparing sensor current responses with clinical glucometer measurements for sweat samples collected before meal, right after meal, and two hours after meal. Reproduced with permission from^115^. Copyright 2020, Elsevier

Porous carbon-based electrochemical sensors represent a significant advancement in sweat analysis technology for real-time biomarker monitoring. Real-time K^+^ concentration monitoring during jogging exercise using portable electrochemical workstations with flexible sensors demonstrates exceptional analytical performance with sensitivity values of 58.6 mV per decade and rapid response times of 0.8 seconds for K⁺ detection (Fig. 12a)^113^. The continuous tracking of potassium ion fluctuations in human sweat shows stable baseline measurements with minimal drift over extended monitoring periods. Mechanical robustness is particularly noteworthy, with devices maintaining 53.2 mV per decade sensitivity after six washing cycles and showing minimal performance degradation during 8000 bending cycles. This durability, combined with negligible potential drift of less than 1.4 mV per hour, renders these sensors suitable for continuous wear applications in athletic performance monitoring and metabolic health assessment.

Porous nanocomposite tactile sensors leverage three-dimensional porous structures to achieve enhanced piezoresistive sensitivity for multimodal physiological monitoring applications. The comprehensive sensor array design and spatial pressure distribution mapping capabilities (Fig. 12b, c) demonstrate the potential for advanced tactile sensing^114^. The fabrication process involves creating interconnected porous networks that combine high elasticity with stable electrical conductivity through synergistic conductive pathways. Tactile sensing performance is demonstrated through real-time pressure mapping, showing distinct response patterns under various loading conditions with high sensitivity of 15 kPa⁻¹ and rapid 100 millisecond response times. The spatial resolution capabilities enable detection of subtle physiological changes, including heart rate variability, arterial pulse patterns, and respiratory dynamics across different finger positions and pressure intensities.

In addition to these tactile and physiological mapping capabilities, porous graphene-based biosensors also show promise for multiplexed biomarker detection in biofluids such as sweat (Fig. 12d)^115^. The flexible, porous graphene network allows simultaneous amperometric tracking of numerous analytes, including glucose, acetaminophen, urea, uric acid, ascorbic acid, potassium chloride, sodium chloride, and lactate, as demonstrated by sequential current response curves under analyte addition. Selectivity analysis confirms robust detection of glucose even in the presence of potential interfering species, confirming suitability for noninvasive metabolic monitoring. Sensor current readouts correlate closely with blood glucose values measured by conventional glucometers, as validated across different time points before and after meal intake. These results highlight the potential of porous materials for real-time, multi-analyte biosensing with direct relevance to personalized healthcare and chronic disease management.

The success of these biomedical applications fundamentally depends upon the unique properties of porous materials, including their high surface-to-volume ratios, tunable pore architectures, and enhanced mass transport characteristics^116–118^. Hierarchical porosity enables efficient analyte diffusion while maintaining mechanical flexibility, and interconnected porous networks facilitate rapid signal transduction and improved sensor responsiveness^119,120^. Furthermore, the ability to functionalize porous surfaces with biorecognition elements and selective membranes opens new possibilities for multiplexed sensing and personalized healthcare monitoring, establishing porous materials as fundamental components of next-generation wearable diagnostic platforms^121–123^.

### Wearable strain sensors

Porous nanocomposite materials have become essential components in next-generation flexible sensor applications owing to their distinctive structural features, which facilitate reliable sensing performance under complex mechanical deformation conditions^124,125^. Hierarchically organized porous networks provide broad pore size distributions spanning microporous to macroporous regimes, while interconnected porosity facilitates efficient stress distribution and simultaneous deformation adaptability^126^.

**Fig. 13:**
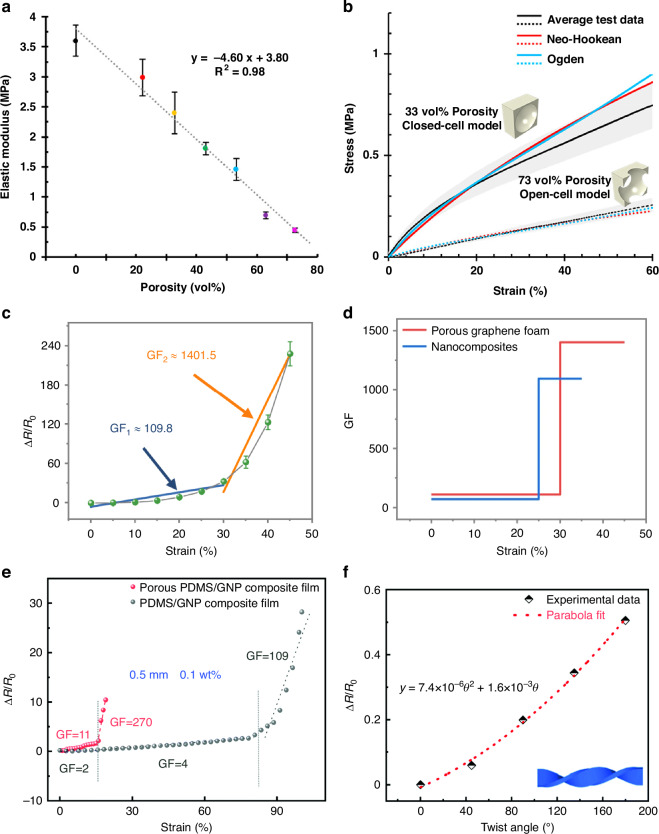
Research on the development of high-performance porous nanocomposites exhibiting property changes upon various mechanical deformations, including stretching, bending, and twisting. **a** Elastic modulus of samples as a function of porosity, with error bars denoting the standard deviation (*n* = 5). **b** Stress–strain curves for ASTM D412 Type C samples with 33 vol% (closed-cell) and 73 vol% (open-cell) porosity; solid lines represent experimental results, while dashed lines correspond to finite element analysis (FEA). Schematics of the unit cell structures for each condition are shown. Reproduced with permission from ref. ^127^. Copyright 2021, American Chemical Society. **c** Relative resistance variation (ΔR/R₀) as a function of applied strain for tensile deformation in the range of 5–45%, with gauge factor (GF) values labeled in the plot. Error bars represent standard deviation (*n* = 3). **d** Gauge factor (GF) of porous graphene foam and nanocomposites as a function of applied strain. Reproduced with permission from ref. ^128^. Copyright 2025, Springer Nature. **e** Relative resistance change (ΔR/R_0_) as a function of tensile strain for both PDMS/GNP and porous PDMS/GNP composite films. **f** Relative resistance change (ΔR/R_0_) as a function of twist angle for a 0.5 mm thick, 0.1 wt% porous PDMS/GNP composite film under torsional deformation. Experimental data are fitted with a parabolic curve. Reproduced with permission from ref. ^130^. Copyright 2022, The Royal Society of Chemistry

Structure-processing-property relationship analysis of 3D-printed porous polymer systems (Fig. 13a) demonstrates systematic changes in elastic modulus with increasing porosity. The elastic modulus decreases linearly from approximately 4 MPa to 0.2 MPa as porosity increases from 0 to 73 vol% (R^2^ = 0.98), with this trend being precisely controllable through direct ink writing (DIW) techniques utilizing sacrificial paraffin fillers^127^. The stress-strain behavior of closed-cell (33 vol%) and open-cell (73 vol%) structures (Fig. 13b) exhibits excellent agreement with both the Neo-Hookean and Ogden models, and the finite element analysis results corroborate the experimental observations.

Strain-dependent piezoresistive response evaluation reveals the exceptional sensing capabilities of porous nanocomposite systems (Fig. 13c, d). Relative resistance change measurements demonstrate progressive increases with applied strain, exhibiting distinct gauge factor transitions at critical deformation points. At moderate strain levels ( ~25%), the system achieves a gauge factor of 109.8, which dramatically increases to 1401.5 at higher strain levels ( ~42%), indicating superior strain sensitivity beyond conventional sensor materials. This multi-regime sensing behavior enables precise detection across broad deformation ranges while maintaining stable electrical response characteristics. The corresponding gauge factor analysis confirms that both porous graphene foam and nanocomposites exhibit stepwise increases above 30% strain, reaching maximum values of 1500, which remains competitive with recently reported CNT-based porous sensors and MXene nanocomposites^128^.

The piezoresistive performance analysis (Fig. 13e) indicates that porous PDMS/GNP composite films exhibit significantly enhanced performance compared to conventional PDMS/GNP films. Particularly noteworthy is the achievement of gauge factor 270 at 65% strain, demonstrating superior performance compared to bulk polymers, porous metal/ceramic systems, and hybrid nanocomposite-based strain sensors^129^. This high sensitivity is comparable to the 57.5% resistance change of SA/CMC composite sponge sensors, reflecting enhanced conductive pathway formation enabled by porous architectures.

The quantitative response characteristics to torsional deformation (Fig. 13f) clearly illustrate the adaptability of porous sensors to complex deformation. A 0.5 mm thick, 0.1 wt% porous PDMS/GNP composite film demonstrates stable parabolic resistance variations (y = 7.4×10⁻⁶θ² + 1.6×10⁻³θ) over a 200° twist angle range^130^, extending beyond the scope of traditional studies focused on uniaxial tension or simple bending. This performance aligns with the torsion resistance of fiber-shaped porous sensors and shear deformation stability of quasi-homogeneous composition sensors, validating their suitability for the multi-axial complex deformation environments required in practical wearable applications^131,132^.

These research findings clearly demonstrate the fundamental advantages of porous nanocomposite materials. Interconnected porous networks enable efficient stress transfer and deformation distribution, while high specific surface areas provide abundant active sites for enhanced sensing performance^133^. Precise control over pore size distributions and surface chemical properties enables tailored responsiveness to specific deformation modes, while well-designed porous frameworks maintain structural integrity under dynamic mechanical conditions.

## Perspectives

Porous material-based sensor technology has achieved remarkable progress, but scalable implementation remains challenged primarily by fabrication process limitations. Achieving uniform and reproducible pore architecture during mass production is complicated by the sensitivity of sol-gel, template-assisted, and 3D printing processes to environmental and operational fluctuations. These process-dependent factors often lead to variability in material quality, device stability, and long-term durability under real-world conditions. Maximizing porosity through advanced processing enhances sensitivity but often compromises mechanical strength and operational reliability.

**Fig. 14:**
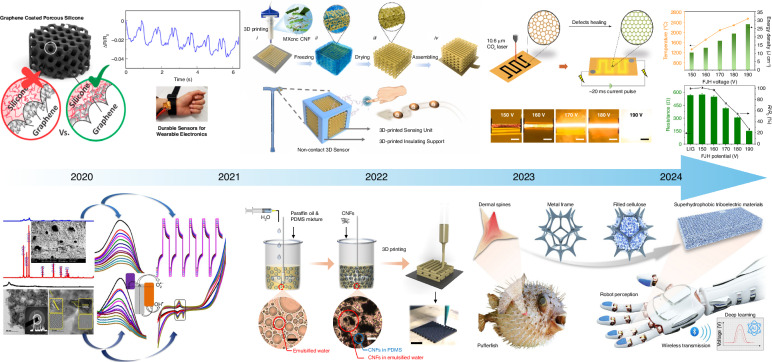
Evolutionary roadmap of porous materials-based sensor technology. Reproduced with permission^134^, Copyright 2020, American Chemical Society; Reproduced with permission^135^, Copyright 2021, Elsevier B.V; Reproduced with permission from ref. ^136^. Copyright 2023, Elsevier B.V.; Reproduced with permission from ref. ^137^. Copyright 2022, Springer Nature; Reproduced with permission from ref. ^138^. Copyright 2024, Springer Nature; Reproduced with permission from ref. ^139^. Copyright 2025, John Wiley & Sons

Recent multi-material and hybrid fabrication strategies, as captured in Fig. 14, reveal persistent obstacles such as module integration, signal crosstalk, and scalability. Cross-sensitivity, thermal management, and signal interference can arise during process-driven miniaturization or multifunctional platform assembly, further restricting device viability^134–139^.

Light-material interaction techniques have shown considerable promise in overcoming many of these challenges. Optical methods such as laser patterning and photonic curing enable precise and non-invasive control over the pore structure and surface properties, enhancing sensor performance by improving sensitivity, selectivity, and durability. Additionally, these light-based processes facilitate faster fabrication and better reproducibility, addressing key limitations inherent in traditional manufacturing approaches.

Future advances should focus on process innovation for integrated, multifunctional sensor platforms, combining nanoscale structural engineering, advanced surface modification, and universal protocols for large-scale, reproducible fabrication. The integration of porous materials with artificial intelligence-driven optimization and IoT platforms, enabled by new fabrication paradigms, will accelerate the realization of robust and practical next-generation sensors^140–142^.

## Conclusion

This review provides a comprehensive examination of recent advances in porous material-based sensor technologies, emphasizing their distinctive advantages including exceptionally high specific surface areas, tunable pore architectures, and extensive chemical versatility. Various categories of porous materials, encompassing metal oxides, polymers, and carbon-based structures, have been systematically engineered through multiple synthesis methodologies including sol-gel processing, template-assisted fabrication, three-dimensional printing, and light-material interactions. The resulting materials have been successfully implemented across diverse sensing mechanisms, including electrical, electrochemical, and optical transduction principles, enabling their deployment in environmental monitoring, biomedical diagnostics, and wearable healthcare systems (Table 1).

**Table 1:** Summary of porous materials characteristics

Porous materials	Structure	Fabrication method	Properties	Application/sensing mechanism		Ref
Metal Oxides	Hierarchical meso/macroporous	Sol–gel Template-assisted	Chemical stability Robust durability	Gas sensing Photodetectors Biosensing	Electrochemical Optical Electrochemical	^43,44,143^
Polymers	Microporous interconnected network Sponge-like structures	3D- printing Template-assisted	Flexibility Elastic compressibility Biocompatibility	Wearable sensors Capacitive/piezoresistive/strain sensors Nitroaromatic compounds detectors	Electrical Electrical Electrochemical	^71,103,108,144^
Carbon-based Materials	3D foam porous graphene Aerogels Nanotube/GO networks	Laser-induced Flashlamp irradiation	Electrical conductivity Mechanical strength	Biosensing Flexible pressure sensors Health monitoring	Electrochemical Electrical Electrical	^17,51,79,145^

The continued advancement in porous material design and fabrication methodologies will facilitate the development of next-generation sensors characterized by enhanced sensitivity, rapid response kinetics, multi-analyte detection capabilities, and robust long-term performance. These technological breakthroughs are anticipated to transform environmental monitoring and personalized healthcare applications by providing sustainable and reliable sensing solutions with unprecedented spatiotemporal resolution and operational flexibility.
